# Interleukin-19 Impairment in Active Crohn’s Disease Patients

**DOI:** 10.1371/journal.pone.0093910

**Published:** 2014-04-09

**Authors:** Elisabet Cantó, Esther Garcia Planella, Carlos Zamora-Atenza, Juan Camilo Nieto, Jordi Gordillo, Ma Angels Ortiz, Isidoro Metón, Elena Serrano, Esteban Vegas, Orlando García-Bosch, Cándido Juárez, Sílvia Vidal

**Affiliations:** 1 Department of Immunology Biomedical Research Institute Sant Pau (IIB Sant Pau), Barcelona, Spain; 2 Department of Digestive Pathology, Hospital de la Santa Creu i Sant Pau, Barcelona, Spain; 3 Department of Biochemistry and Molecular Biology, Faculty of Pharmacy, University of Barcelona, Barcelona, Spain; 4 Bioinformatics Platform, PSCT Platforms, Biomedical Research Institute Sant Pau (IIB Sant Pau), Barcelona, Spain; 5 Department of Statistical, Faculty of Biology, University of Barcelona, Barcelona, Spain; 6 Department of Digestive, Hospital de Sant Joan Despí Moisès Broggi, Sant Joan Despí, Barcelona, Spain; 7 Department of Immunology, Hospital de la Santa Creu i Sant Pau, Barcelona, Spain; 8 Universitat Autònoma de Barcelona, Barcelona, Spain; University of Chicago, United States of America

## Abstract

The exact function of interleukin-19 (IL-19) on immune response is poorly understood. In mice, IL-19 up-regulates TNFα and IL-6 expression and its deficiency increases susceptibility to DSS-induced colitis. In humans, IL-19 favors a Th2 response and is elevated in several diseases. We here investigate the expression and effects of IL-19 on cells from active Crohn’s disease (CD) patient. Twenty-three active CD patients and 20 healthy controls (HC) were included. mRNA and protein IL-19 levels were analyzed in monocytes. IL-19 effects were determined in vitro on the T cell phenotype and in the production of cytokines by immune cells. We observed that unstimulated and TLR-activated monocytes expressed significantly lower IL-19 mRNA in active CD patients than in HC (logFC = −1.97 unstimulated; −1.88 with Pam3CSK4; and −1.91 with FSL-1; p<0.001). These results were confirmed at protein level. Exogenous IL-19 had an anti-inflammatory effect on HC but not on CD patients. IL-19 decreased TNFα production in PBMC (850.7±75.29 pg/ml vs 2626.0±350 pg/ml; p<0.01) and increased CTLA4 expression (22.04±1.55% vs 13.98±2.05%; p<0.05) and IL-4 production (32.5±8.9 pg/ml vs 13.5±2.9 pg/ml; p<0.05) in T cells from HC. IL-10 regulated IL-19 production in both active CD patients and HC. We observed that three of the miRNAs that can modulate IL-19 mRNA expression, were up-regulated in monocytes from active CD patients. These results suggested that IL-19 had an anti-inflammatory role in this study. Defects in IL-19 expression and the lack of response to this cytokine could contribute to inflammatory mechanisms in active CD patients.

## Introduction

IL-19 is mainly produced by myeloid cells, B cells and epithelial cells. It can be induced by the TLR4 ligand (LPS) and granulocyte-macrophage colony-stimulating factor (GM-CSF). Priming monocytes with IL-4 or IL-13 but not IFNγ significantly increases the levels of IL-19 mRNA induced by subsequent stimulation with LPS [1]. IL-19 functions through a receptor complex consisting of IL-20Rα and IL-20Rβ that is also used by IL-20 and IL-24 [2]. High levels of both receptor chains are present in several tissues, including skin, lung, heart, small intestine, salivary glands, and testes. Expression of the IL20Rα chain has not been detected on human leukocytes to date, suggesting that immune cells are only a minor target for the effects of IL-19 [3]. However, these results are not conclusive because other studies have shown that IL-19 enhances the production of Th2 cytokines in T cells [4] and IL-10 but not TNFα in unstimulated monocytes [5]. In addition, it has been reported the effects of IL-19 on human and mouse immune cells differ because it induces IL-6 and TNFα in mice but not in humans [6]. Recently, Ching-Hua Yeh et al. reported that the serum levels of IL-19 correlate inversely with the levels of IFNγ-secreted CD4+T cells after PMA/ionomycin stimulation. These authors showed that IL-19 up-regulates Foxp3 mRNA and induces the regulatory function of human CD4+ T cells. Altogether these results suggest that IL-19 plays an intriguing role during the immune response [7].

IL-19 is elevated in asthma patients [8] and in uremic patients [9]. It is also involved in the pathogenesis of psoriasis [10], [11], neoplastic processes [12], [13], endotoxic shock [14], chronic rhinosinusitis [15], vascular disease [16], [17], [18], and rheumatoid arthritis [19], [20], [21]. Regarding IL-19 effects in vivo, Azuma et al. [22] reported that IL-19-deficient mice are more susceptible to experimental acute colitis induced by dextran sodium sulfate (DSS) than wild type (WT) mice. Crohn’s disease (CD) is an inflammatory bowel disease (IBD). Its etiopathogenesis remains poorly understood, but it includes genetic susceptibility, enteric environmental factors and immunological responses. It is characterized by an imbalanced activation of Th1 and Th2 dependent immune responses. Activated cells from CD patients produce higher levels of IFNγ, IL-17 and TNFα [23], [24]. Increased expression of intracellular IL-2, IL-6, and IL-10 in active Crohn’s disease patients has also been reported [25]. Besides, a central immunological imbalance favors the development of disease in mice [26]. Knockout mice for IL-2 [27] and IL-10 [28] spontaneously develop colitis, and it was recently documented that IL-19-deficient mice present increased susceptibility to acute DSS-induced colitis. This increased susceptibility correlated with the accumulation of macrophages and the increased expression of many different cytokines (IFNγ, IL-1β, IL-6, IL-12 and TNFα). Bone marrow derived macrophages from IL-19-deficient mice produced higher levels of TNFα, IL-6 and IL-12 than WT mice after LPS stimulation, suggesting a role for IL-19 in the innate response to microbial stimuli.

In the present study we aimed to address the expression, regulation and effect of IL-19 in active CD patients. We determined mRNA, protein levels of IL-19 and the effect of this cytokine on immune cell cultures from active CD patients and healthy controls (HC).

## Materials and Methods

### Peripheral Blood Samples

Peripheral blood samples were collected from healthy controls and active CD patients.

Written informed consent was obtained from all participants and ethical approval for the study was granted by the Hospital de la Santa Creu i Sant Pau Institutional Ethics Committees. Institutional Ethics Committee approved this consent procedure. Twenty-three patients with CD attending the IBD outpatient clinic were included in the study. Table 1 shows the demographic and clinical relevant data of the CD enrolled patients according to Montreal classification for age at onset; disease location and behaviour. Twenty age- and sex-matched HC were also evaluated. CD diagnoses were based on clinical, radiologic, endoscopic and pathological bases. All of the patients had active disease at the time of blood collection. To avoid the influence of treatment in our analysis, we selected a cohort of active CD patients who were not receiving immunomodulators in the 6 months prior to blood collection. Peripheral blood mononuclear cells (PBMC) were isolated by Ficoll-Hypaque (Lymphoprep, Axis-Shield PoC As, Oslo, Norway) density gradient centrifugation. Cells were washed twice, resuspended in FCS+10% DMSO, placed in cryogenic vials and kept in liquid nitrogen until needed.

**Table 1:** Demographic and clinical characteristics of patients included in this study.

	Active CD patients
Age (year):	43.59±15.92
Sex:	Men: 37.5%
	Women: 62.5%
Age at diagnosis:	A1 (below 16 years) = 0%
	A2 (between 17–40 years) = 82.6%
	A3 (above 40) = 17.4%
Location:	L1 (ileal) = 39.2%
	L2 (colonic) = 47.8%
	L3 (ileocolonic) = 13.0%
Behaviour:	B1 (non-stricturing) = 100%
Activity:	Mild: 30%
	Moderate: 52.1%
	Severe: 17.3%
Perianal:	Yes: 22.2%
	No: 77.78%
Surgery:	Yes: 24.3%
	No: 75.7%
Smoke:	Yes: 60%
	No: 40%

### Purification of Monocytes

Monocytes were obtained from PBMC by negative selection using the Human Monocyte Enrichment KIT (EasySep StemCell, Grenoble, France). The viability of recovered non-touched population was determined by Trypan Blue (>99%). The purity of monocytes was evaluated by flow cytometry and was always >95%.

### Genotyping of TLR1 Variants

As TLR1 polymorphism can influence cytokine production after Pam3CSK4 activation [29], *TL*R1 602 alleles *(I/S, I/I, S/S)* were determined in genomic DNA extracted from blood samples using Qiagen kit (Qiagen, Heiden, Germany). The primers used were 5′ AGGGGACAATCCATTCCAAT 3′ and 5′ GGGCACGATTCTTTCTGGG3′. Once the amplification was confirmed, sequencing was performed by Mocrogen Inc, Korea using BigDye (Applied Biosystems, Life Technologies, Camarillo CA, USA) chemistry.

### Cell Culture

PBMC or purified monocytes were cultured (1×10^6^ cells/ml) in complete RPMI 1640 medium (BioWhittaker, Verviers, Belgium) including 10% fetal calf serum, 2 mM glutamine, 100 U/ml penicillin and 100 μg/ml streptomycin (Biowhittaker) and with TLRs agonists: TLR2/TLR6 agonist, FSL-1 (0.01 μg/ml), TLR2/TLR1 agonist, Pam3CSK4 (0.1 μg/ml) and TLR4 agonist, LPS (0.2 μg/ml) (Invivogen, San Diego, CA). To analyze the effect of exogenous IL-19, PBMC were treated with rhIL-19 (400 ng/ml) (ImmunoTools, Friesoythe, Germany). To study the effect of IL-10 on IL-19 production, rhIL-10 (200 ng/ml) (Preprotech, USA) and blocking anti-IL-10 (500 ng/ml) (BD, Biosciences, USA) were added to PBMC cultures.

### Measurement of Cytokine Concentration

IL-19 content in serum and after TLR activation was determined using Quantikine Human IL-19 Immunoassay (R&D systems. Minneapolis MN USA). Supernatants from cell cultures were tested for TNFα, IL-10, IFNγ (BD) and IL-4 (Immunotools) content by specific ELISA, according to the manufacturer’s instructions, using the quantitative colorimetric sandwich ELISA kit.

### T cell Activation and Expansion

PBMC from active CD patients and HC were activated and expanded using T cell Activation Expansion Kit (Milteny Biotec. Germany). Cultures were performed with or without rhIL-19 (400 ng/ml), and after 3 and 9 days of culture, cells were removed and their phenotype was analyzed by flow cytometry.

### Flow Cytometry

To analyze T cell phenotype after 3 and 9 days of activation and expansion culture, cells were removed and labeled with anti-CD4-PECy7 (Biolegend, San Diego, CA, USA), anti-CD25-FITC (Immunotools), anti-CD127-Alexa647 (BD), and anti-CTLA4-PE (BD) or anti-GITR-PE (BioLegend) or the corresponding isotype control Igs. Stained cells were analyzed by flow cytometry with Cytomics FC 500 (Beckman Coulter, Miami, FL).

### RNA Isolation

Total RNA from purified monocytes cultured with medium or with Pam3CSK4 and FSL-1 ligands was purified using the Mini Qiagen Kit RNA (Hilden, Germany). RNA was resuspended in RNase-free H_2_O and stored at −80°C until use. RNA concentration was determined using a NanoDrop spectophotometer (Thermo Fisher Scientific, Wilmington, DE, USA) and RNA integrity number (RIN) was analyzed using a RNA 6000 Nano LabChip Kit (Agilent Technologies, Boeblingen, Germany) with an Agilent 2100 Bioanalyzer according to the manufacturer’s instructions. All samples used in this study had a RIN>9.0.

### Microarray Hybridization and Data Analysis

Microarray analysis was performed using Affymetrix GeneChip Human Gene U133A 2.0 Array (HG-U133A) (Affymetrix, Santa Clara, CA, USA) according to the manufacturer’s instructions. Briefly, isolated RNA (100 ng) was reverse transcribed to double-stranded cDNA. In vitro transcription synthesis of aRNA was performed at 40°C during 16 h. In all cases, 12 μg of each aRNA preparation was fragmented and hybridized onto a GeneChip Human Gene U133A 2.0 Array for 16 hours in a Hybridization Oven 640 (Affymetrix). The arrays then were washed and stained on a GeneChip Fluidics Station 450 (Affymetrix). Scanned images were converted to CEL files by GeneChip Command Console Software (AGCC) (Affymetrix). Raw expression values were pre-processed using the Robust Multiarray Averaging method. These normalized values were used for all subsequent analyses. Data were subjected to non-specific filtering to remove low signal and low variability genes. The selection of differentially expressed genes was based on a linear model analysis with empirical Bayes modification for the variance estimates. p-values were adjusted to obtain a strong control over the false discovery rate using the Benjamini-Hochberg method.

### Quantification of miRNA Expression Level

Total RNA enriched with small RNAs was obtained from purified monocytes from HC and active CD patients using mirVana miRNA Isolation Kit (Ambion, Invitrogen, Life Technologies). The small RNA fractions were reverse transcribed using Megaplex RT primers and the expression level of 384 miRNAs was analysed using TaqMan MicroRNA Arrays (Array A) (Applied Biosystems, Life Technologies). Experimental data were analyzed by *DataAssist* Software (Life Technologies). The relative miRNA expression values were calculated using RNU44 as endogenous control.

### Real Time RT-PCR

Using specific microRNA primers, cDNA from RNA was synthesized with TaqMan MicroRNA Reverse Transcription Kit (Applied Biosystems, Life Technologies). Real-Time PCR was performed from 1.33 μl of cDNA products using TaqMan Universal PCR Master Mix, no UNG and the TaqMan Small RNA assays. All expression levels were normalized to an endogenous control RNU44. Experimental data were analyzed by *DataAssist* Software (Life Technologies).

### Statistical Analysis

Statistical analyses were performed with GraphPad Prism 5.0 (GraphPad Software Inc. San Diego CA). Data are presented as mean ± SEM. Differences were determined by student’s unpaired t-test or the Mann-Whitney U-test and paired t test or Wilcoxon matched pairs test when samples are related, depending on Gaussian approximation.

## Results

### Impaired IL-19 Gene Expression in Monocytes from Active CD Patients

**Figure 1:**
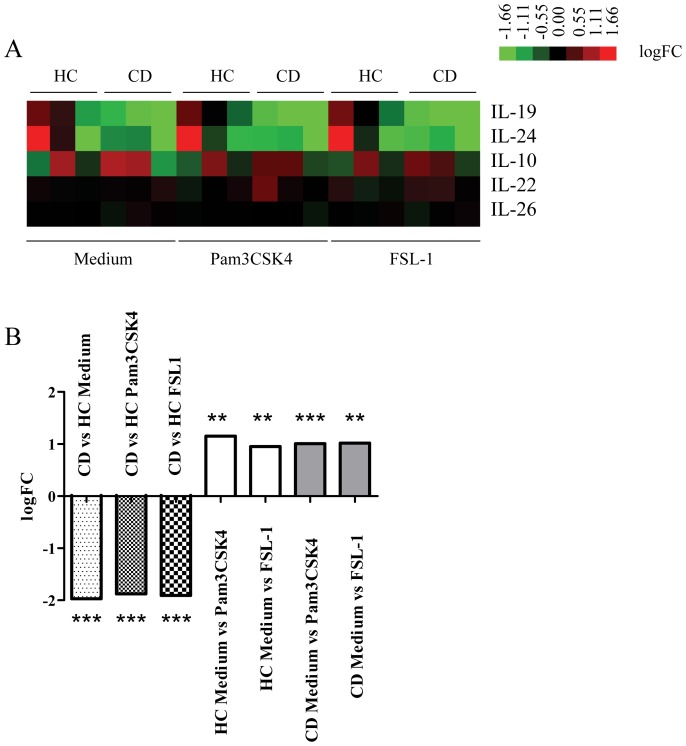
Heat map image of IL-10 family cytokines. Monocytes mRNA was extracted from HC (n = 3) and active CD patients (n = 3) after 6 h of culture with medium in the absence or presence of Pam3CSK4 and FSL-1. A) Green denoted down-regulated and red up-regulated genes. B) logFC of IL-19 mRNA expression between CD and HC are shown. **p<0.01; ***p<0.001.

In a microarray analysis of purified monocytes heterozygous for the TLR1 SNP I602S, we identified genes that were differentially down-regulated in monocytes from active CD patients compared to monocytes from HC (53 genes in monocytes cultured with medium; 78 genes in monocytes cultured with Pam3CSK4 and 60 genes in monocytes cultured with FSL-1) (data not shown). Among down-regulated genes in monocytes from active CD patients, we found IL-19, a cytokine related to the IL-10 family (Fig. 1A). We observed significant differences in the mRNA levels for IL-19 between CD patients and HC after 6 h of culture in medium and after TLR activation. IL-19 mRNA in CD monocytes was significantly lower than in HC monocytes in all conditions assayed. As shown in Figure 1B, after 6 h of culture, the logFC in IL-19 expression between monocytes isolated from active CD patients vs HC was: −1.97 with medium (p<0.001); −1.88 with Pam3CSK4 (p<0.001) and −1.91 with FSL-1 (p<0.001). After TLR2 activation, monocytes from active CD patients and HC increased IL-19 mRNA levels (logFC 1.01, p adjusted <0.01 after Pam3CSK4 activation, logFC 1.017 p adjusted <0.01 after FSL-1 activation).

### TLR Activation Increases the Production of IL-19 in Monocytes and PBMC

**Figure 2:**
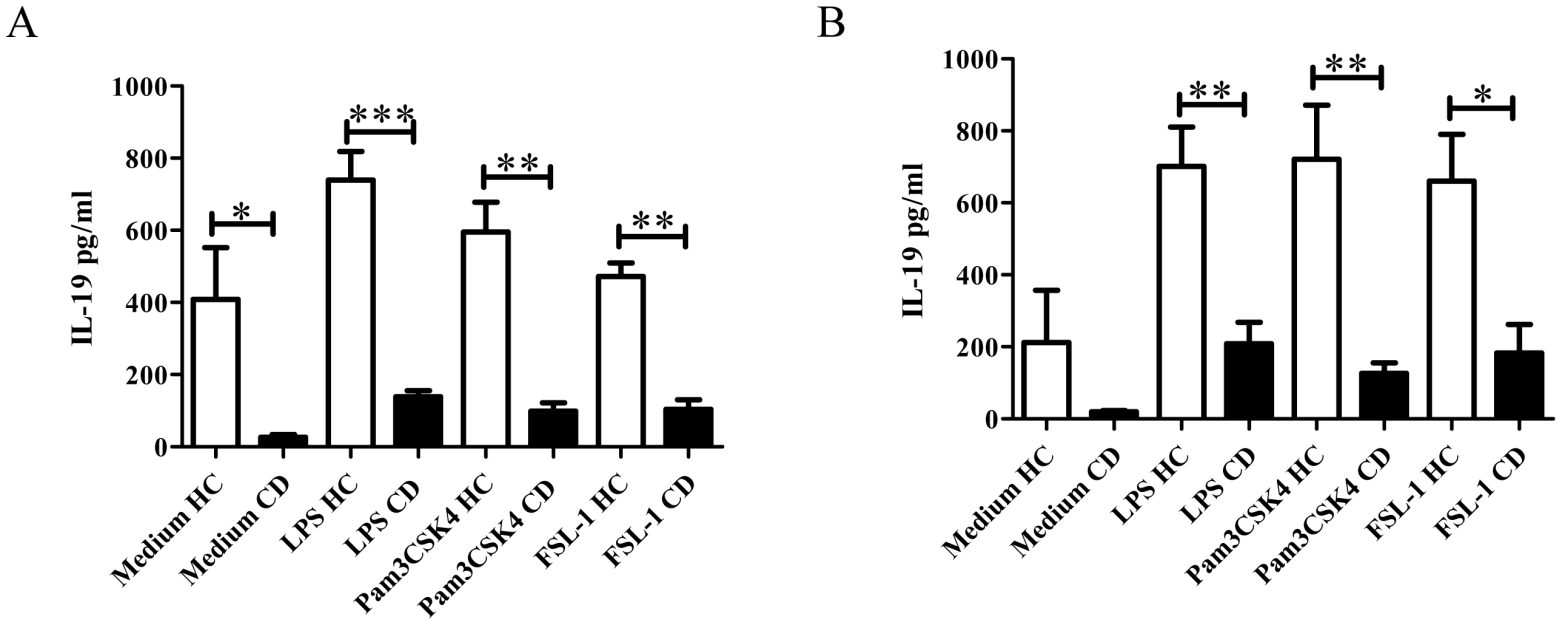
IL-19 production after TLR activation in PBMC cultures from active CD patients (n = 3) and HC (n = 6). PBMC were cultured 24(A) and 48 h (B) with different TLR ligands (LPS, Pam3CSK4 or FSL-1) and IL-19 production was determined by ELISA as described in Materials and Methods. The viability of PBMC was determined by Trypan Blue (>99%). *p<0.05; **p<0.01; ***p<0.001.

To analyze IL-19 at protein level purified monocytes and PBMC from active CD patients and HC were cultured for 24 h and 48 h in the presence of TLRs ligands. Purified monocytes from active CD patients cultured with medium released lower amounts of IL-19 than HC after 24 h of culture (345±306 pg/ml vs 1769±1026 pg/ml, respectively). After TLR stimulation, both types of cells increased the release of IL-19 (CD: LPS: 568±622 pg/ml; Pam3CSK4∶740±819 pg/ml; FSL-1∶980±991 pg/ml vs HC: LPS: 2433±216 pg/ml; Pam3CSK4∶2558±169 pg/ml; FSL-1∶2284±38 pg/ml). To determine whether lymphocytes alter IL-19 production by monocytes, we cultured PBMC from active CD patients and HC. At 24 h of culture with medium, PBMCs from CD produced significantly lower levels of IL-19 (CD: 26±7.63 pg/ml of IL-19 vs HC: 408±143.1 pg/ml of IL-19 (p<0.05) (Fig. 2A). After TLR activation with TLR4 and TLR2 ligands, PBMC from active CD patients also secreted significantly lower amounts of IL-19 than PBMC from HC (LPS: 150.3±17.53 vs 606.7±39.53; p<0.001; Pam3CSK4∶147.0±50.82 vs 595.8±81.85; p<0.01; FSL-1∶101.0±19.15 vs 472.0±37.83; p<0.001). Similar results were obtained after 48 h of culture with medium and in the presence of TLR ligands (Fig. 2B) (CD: medium: 19.5±3.5 pg/ml; LPS: 208.7±59.38 pg/ml; Pam3CSK4∶126.3±28.75 pg/ml; FSL-1∶183.0±79.1 pg/ml vs HC: medium: 212±145 pg/ml; LPS: 701±109.1 pg/ml; Pam3CSK4∶721.0±150 pg/ml; FSL-1∶660.7±129.9 pg/ml). Consistent with a decreased production of IL-19 in monocytes and PBMC from active CD patients, the content of total circulating IL-19 showed a tendency to decrease in serum of active CD patients (CD: 19.03±8.06 pg/ml vs HC: 28.27±10.27 pg/ml) (data not shown).

### rhIL-19 Decreases TNFα Production

**Figure 3:**
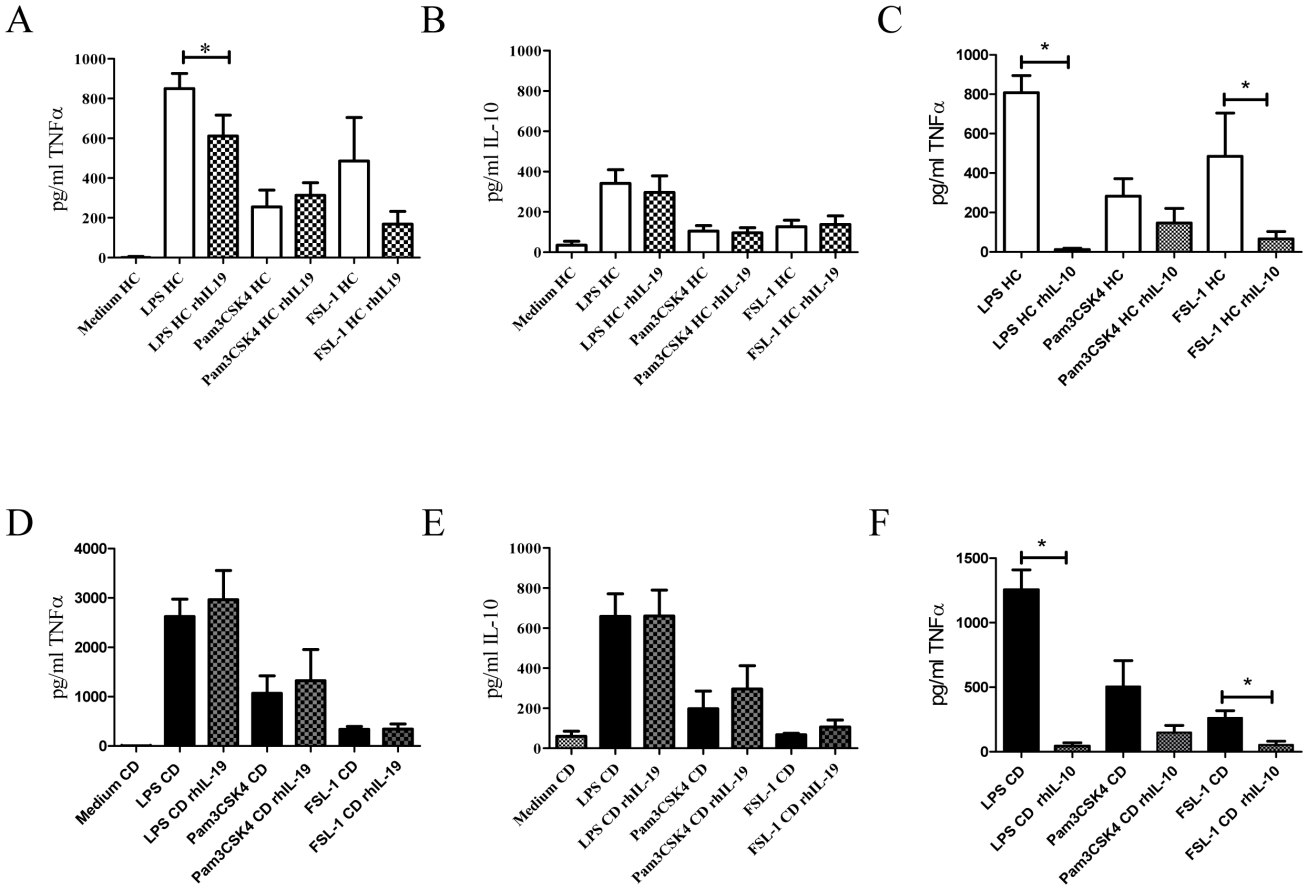
rhIL-19 down-regulates TNFα production in cultures from HC after LPS activation. PBMC from HC and active CD patients were cultured with LPS (HC n = 10 and CD n = 5), Pam3CSK4 (HC n = 10 and CD n = 5) and FSL-1 (HC n = 5 and CD n = 3) for 24 h with or without rhIL-19 (400 ng/ml). A and B) TNFα and IL-10 production in cultures from HC after TLR activation with or without rhIL-19. D and E) TNFα and IL-10 production in cultures from active CD patients after TLR activation with or without rhIL-19. C and F) TNFα production from HC and active CD patients cultured with rhIL-10 (200 ng/ml). *p<0.05.

To analyze functional consequences of the decreased IL-19 production in active CD patients, we added exogenous IL-19 in PBMC cultures. PBMC from active CD patients and HC were activated with TLR4 and TLR2 ligands for 24 h in the presence or absence of rhIL-19. Supernatants were collected and TNFα and IL-10 were determined. As expected from our previous work [23] after LPS and Pam3CSK4 activation, PBMCs from CD patients produced higher amounts of TNFα than HC (2626.0±350 pg/ml vs 850.7±75.29 pg/ml, p<0.01 and 1068.0±355.6 pg/ml vs 254.0±84.54 pg/ml p<0.05). In contrast, after FSL-1 activation, we observed a similar TNFα production in CD and HC PBMCs (337.0±59.99 pg/ml vs 485.8±218.9 pg/ml). After LPS activation, PBMCs from active CD patients produced higher amounts of IL-10 than HC (658.8±112.7 pg/ml vs 341.0±68.19 pg/ml p<0.05). However, after TLR2 activation, PBMCs from active CD patients and HC produced similar amounts of IL-10 (Fig. 3). We could not confirm that differences in TNFα production were partly associated with the percentage of monocytes in these samples. However, in our previous results [23] no significant differences in the percentage of monocytes in PBMC was observed between active CD patients and HC.

When we added rhIL-19 to cell cultures, the pattern of cytokine production changed. Addition of rhIL-19 significantly decreased TNFα production in LPS cultures of PBMC from HC (850.7±75.29 pg/ml vs 610.9±105.1, p<0.05), but not from active CD patients (2626.0±350 pg/m vs 2965.1±588.5 pg/m) (Fig. 3A and 3D). Addition of rhIL-19 induced no changes in TNFα production after TLR2 activation and no changes in IL-10 after TLR2 or TLR4 activation. (Fig. 3B and 3E).

To compare the effect produced by IL-19 and IL-10 on TNFα production, PBMC were cultured in the presence of rhIL-10. Addition of IL-10 significantly decreased TNFα production in LPS cultures (CD: 1255.0±153.6 pg/ml vs 44.0±25.4 pg/ml, p<0.01; HC 808±86.16 pg/ml vs 12.0±6.9 pg/ml, p<0.01) and in FSL-1 cultures (CD: 261.3±56.26 pg/ml vs 53.67±28.6 pg/ml, p<0.05; HC: 485.8±218.9 pg/ml vs 66.6±37.0 pg/ml, p<0.05) (Fig. 3F and 3C).

### IL-19 Up-regulates IL-4 on Activated T Cells

**Figure 4:**
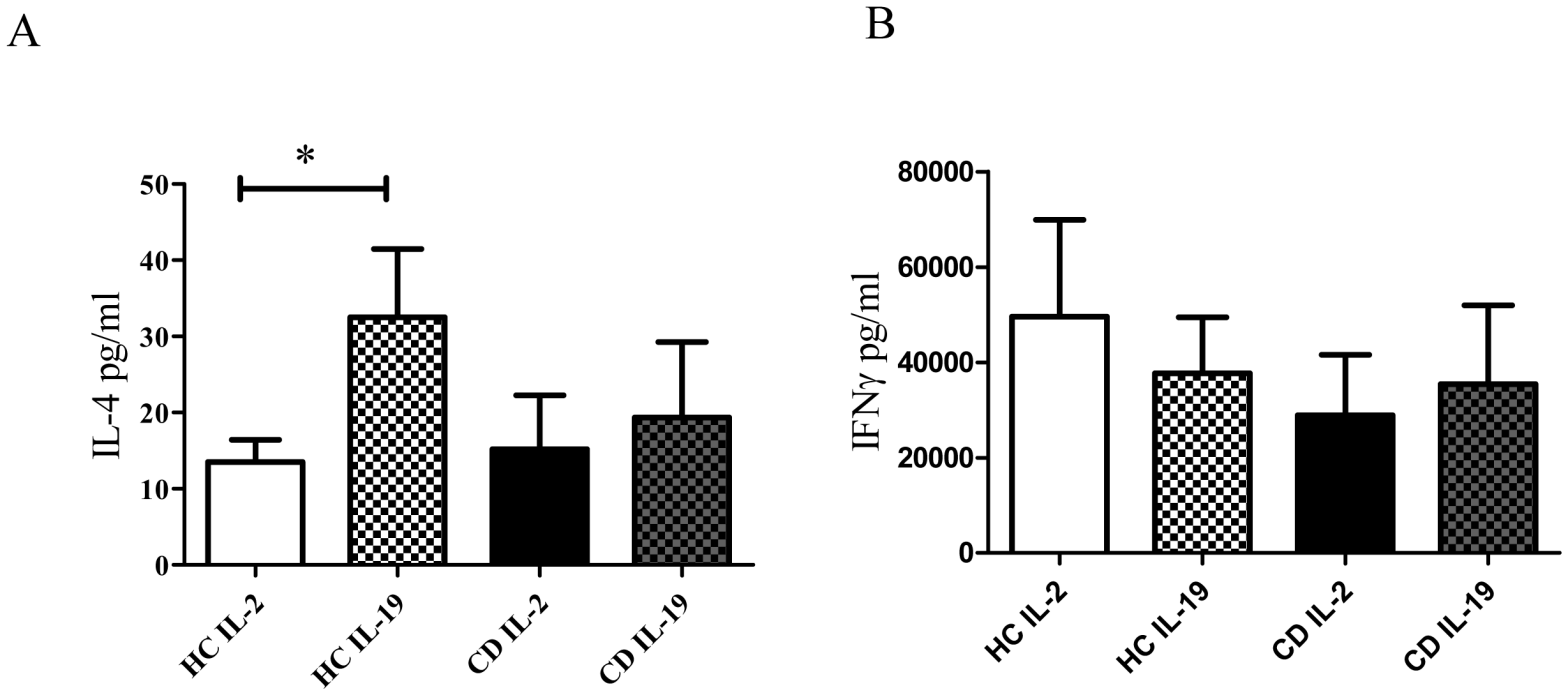
rhIL-19 up-regulates IL-4 production in activated T cells. PBMC from HC (n = 6) and active CD (n = 6) were activated and expanded with anti-CD3+anti-CD2+anti-CD28 as described in Materials and Methods. Supernatants were removed and IL-4 and IFNγ content were analyzed by ELISA. A) A) Up-regulation of IL-4 by rhIL-19 in HC cultures. B) IFNγ content in HC and CD patients in the presence or absence of rhIL-19.*p<0.05.

To study the effect of rhIL-19 on T cells, long exposure cultures to IL-19 were performed [4], [8]. Similar percentage of CD4+ cells were observed in PBMC from active CD patients and HC (% of CD4+ cells expressed as mean ± SD: 27.77±12.21 and 22.23±8.91, respectively; n = 8; *p* = 0.3178) before culture. T cells from active CD patients and HC were activated and expanded for 9 days. Supernatants were removed and cytokine content was determined. As shown in Figure 4, after 9 days of culture, the addition of rhIL-19 to HC cultures increased IL-4 production (HC: 32.5±8.9 pg/ml with IL-19 vs 13.5±2.9 pg/ml without IL-19, p<0.05) whereas IL-4 content remained unaffected in active CD cultures (15.17±7.0 pg/ml with IL-19 vs 19.33±9.9 pg/ml without IL-19). Normalized values of IL-4 production in relation to the % of CD4+ cells also indicated that IL-9 was able to increase IL-4 production in PBMC cultures from HC but not from active CD patients (HC: 0.69±0.21 with IL-2 and 1.81±0.61 with IL-19; p<0.05; active CD patients: 0.33±0.85 with IL-2 and 0.58±0.313 with IL-19). No changes in IFNγ content were observed when rhIL-19 was added to cultures of active CD patients or HC.

### IL-19 Up-regulates CTLA4 Expression on CD4+ T Cells

**Figure 5:**
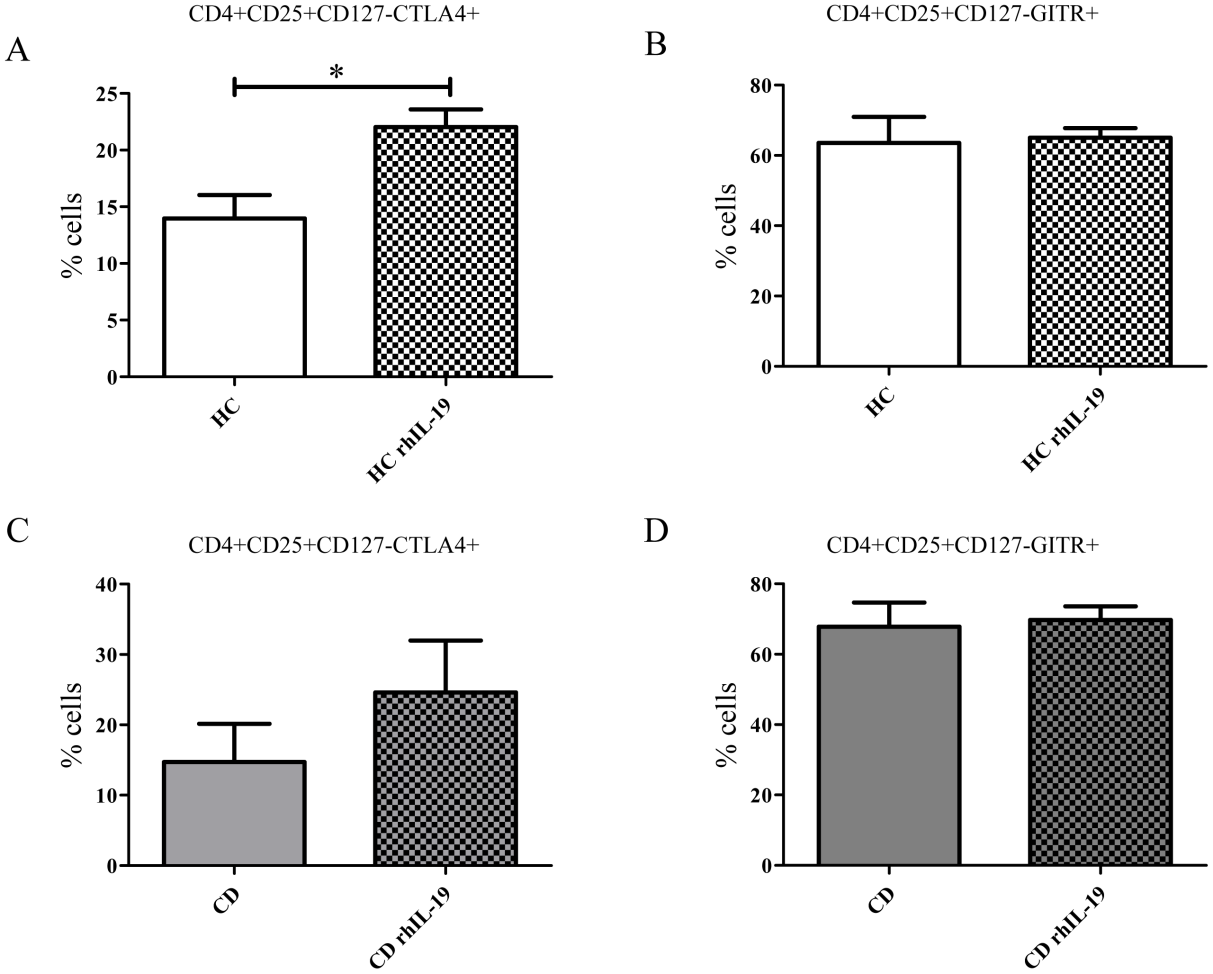
rhIL-19 up-regulates CTLA4 expression on CD4+CD25+CD127 − **cells.** PBMC from HC (n = 4) and active CD (n = 3) were activated with anti-CD3+anti-CD2+anti-CD28 as described in Materials and Methods. The expression of CTLA4 (A) and GITR (B) was determined on CD4+CD25+CD127− cells by flow cytometry. *p<0.05.

A recent report suggested that IL-19 affects the regulatory activity of CD4+ T cells from HC [7]. To study whether long exposure to IL-19 can modify CD4+ phenotype in active CD patients we performed PBMC cultures with and without rhIL-19. We analyzed the expression on CD4+CD25+CD127− cells of cytotoxic T-lymphocyte antigen 4 (CTLA4) and glucocorticoid-induced TNFR-related protein (GITR) as markers to characterize regulatory cells in CD4+CD25+CD127− cells [30]. Before culturing, no expression of CTLA4 or GITR was detected on CD4+CD25+CD127− cells. After 3 days in culture, a low percentage of CD4+CD25+CD127− expressed CTLA4 but no GITR from both active CD patients and HC (data not shown). After 9 days, rhIL-19 increased the percentage of CD4+CD25+CD127−CTLA4+ cells from active CD patients (24.61±7.38% vs 14.70±5.43%; p = 0.09) and HC (22.04±1.55% vs 13.98±2.05%; p<0.05) (Fig. 5A and 5C). No differences were observed on GITR expression in cell cultures from either active CD patients or HC in the presence or absence of rhIL-19 (67.86±6.86% vs 69.75±3.9% for active CD and 63.58±7.3% vs 65.06±2.72% for HC) (Fig. 5D and 5B).

### IL-10 Regulates IL-19 Production

**Figure 6:**
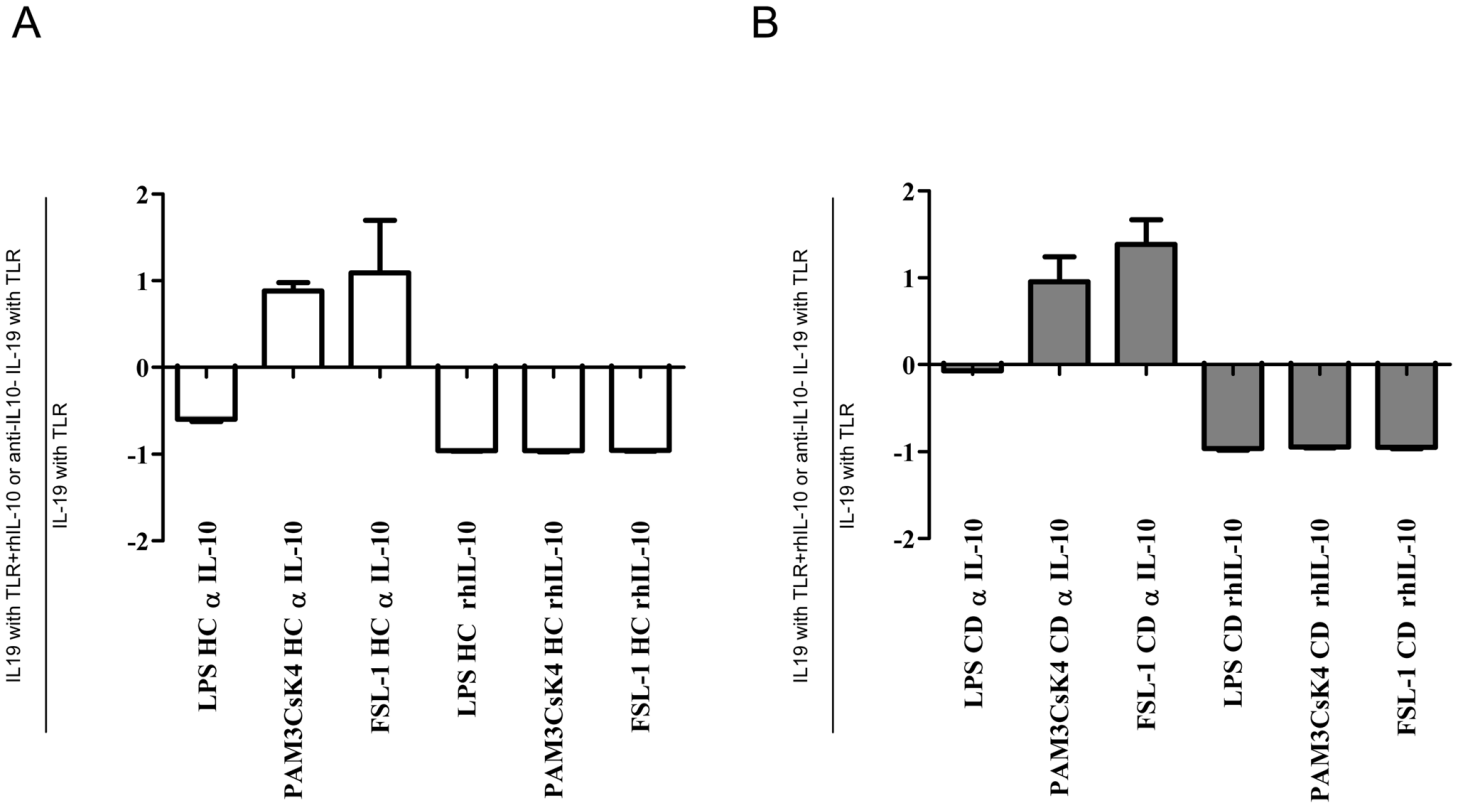
IL-10 regulates IL-19 expression. PBMC from A) HC (n = 3) and B) active CD patients (n = 3) were activated 24 h with TLR ligands (LPS, Pam3CSK4 and FSL-1) in the presence of rhIL-10 (200 ng/ml) and blocking anti-IL10 (500 ng/ml). Results are expressed as: (IL19 production with TLR+rhIL-10 or anti-IL10-IL-19 production with TLR)/IL-19 production with TLR.

J Williams et al 2005 [5] observed that IL-10 decreased IL-19 mRNA expression in unstimulated monocytes from HC. To analyze whether IL-10 can regulate IL-19 production in active CD patients after TLR activation, we cultured PBMC from active CD patients and HC with TLR ligands in the presence or absence of rhIL-10. In all assays, IL-19 production decreased when rhIL-10 was added to cultures. Furthermore, IL-19 production increased similarly in active CD patients and HC when anti-IL-10 blocking antibodies were added to the Pam3CSK4 and FSL-1 cultures but not to LPS cultures (Fig. 6A and 6B). IL-19 production was similarly regulated by IL-10 in HC and CD patients.

### Three miRNAs Targeting IL-19 are Up-regulated in Active CD Patients

**Figure 7:**
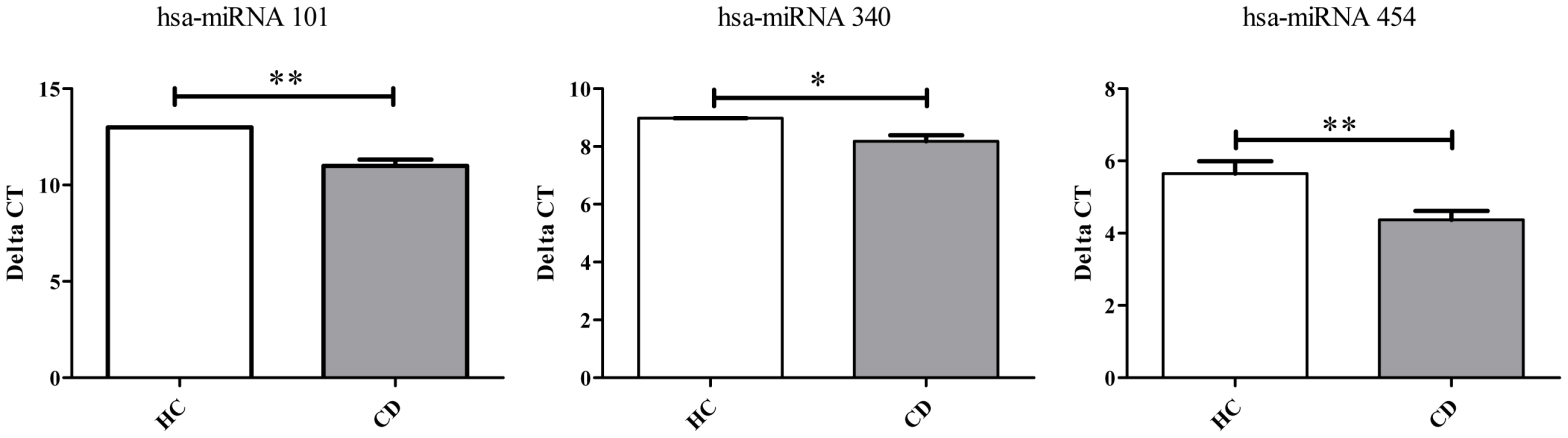
Three miRNAs targeting IL-19 are up-regulated in active CD patients. miRNA expression in purified monocytes from HC (n = 3) and active CD patients (n = 5) were determined by RT-PCR as described in Materials and Methods. Different expression is shown as Delta Ct values. **p<0.01; ***p<0.001.

We next analyzed whether the lower levels of IL-19 mRNA in CD patients were a consequence of the up-regulation of specific miRNAs targeting IL-19. To this end, purified monocytes were cultured and after 3 h, RNA enriched with miRNA was extracted and the expression of 384 miRNAs was analyzed. Computational prediction of IL-19 mRNA targets for miRNAs was carried out with Microcosm Targets (http://www.ebi.ac.uk/enright-srv/microcosm/htdocs/targets/v5/) [31]. Silico analysis let us to identify statistically significant (p<0.05) potential binding sites in the IL-19 mRNA for three up regulated miRNAs in monocytes from active CD patients: hsa-miRNA 101 (at position 302–322, score 15.5693), hsa-miRNA 340 (at position 70–91, score 16.6028), and hsa-miRNA 454 (at position 298–320, score 18.1842) (Fig. 7).

## Discussion

We found that active CD patients had an additional defect in the regulation of the immune response. Monocytes from active CD patients had lower IL-19 expression than those from HC. In addition, PBMC from CD did not respond to IL-19. On the contrary, cells from HC responded to IL-19, decreasing TNFα and increasing IL-4 production and CTLA4 expression. This response in HC confirms the anti-inflammatory role of IL-19.

Low IL-19 expression and production may contribute to CD etiopathology. This possibility is supported by the experiments of Azuma et****al who showed that IL-19^−/−^ mice have higher susceptibility to acute DSS-induced colitis than wild-type mice. Their deficient mice expressed higher amounts of IL-6, TNFα and IL12 but no change in IL-10 after TLR4 activation [22]. Similarly, we showed that monocytes from active CD patients produced more TNFα than healthy donors in response to TLRs.

Our findings suggest two regulatory mechanisms to explain the low IL-19 expression in active CD patients. One mechanism could be the regulation at gene transcription. IL-19 lies in a cluster of four IL-10 family member genes. The IL-19 promoter contains binding motifs for keratinocyte growth factor (FGF7), two NF-kB sites and two CAAT enhancer binding protein sites. Key elements of control over the IL-19 promoter are an AML1 site (RUNX1) and the STAT6 sites [32]. Interestingly, unpublished data from our microarray analysis showed that monocytes from active CD patients express lower levels of STAT6 and FGF7 than monocytes from HC. Another mechanism could be the epigenetic regulation by miRNAs. miRNAs are described as small noncoding RNAs that regulate gene expression by binding to complementary target mRNAs, promoting mRNA decay or inhibiting mRNA translation [33]. Based on computational prediction we observed that three miRNAs that can modulate IL-19 mRNA expression (hsa-miRNA 101, hsa-miRNA 340 and hsa-miRNA 454) were up-regulated in monocytes from active CD patients (Fig. 7). Consistent with our observation, mirRNA-101 was up-regulated in the peripheral blood lymphocytes of an experimental model of TH1-mediated inflammatory bowel disease [34]. We cannot rule out other regulatory mechanisms such as the methylation status of IL-19 gene. However, this is a controversial issue. One study reported that the IL-19 gene was hypomethylated in whole blood from ileal inactive CD female patients when compared to HC [35]. However, another study found that the murine IL-19 gene was methylated in CD4+T lymphocytes, and as a result, IL-19 was not transcribed in T cells from these mice [36]. Taken together, these data suggest that IL-19 expression is tightly regulated. Further studies are needed to clarify the mechanism that restrains IL-19 mRNA expression in monocytes from active CD patients.

The exact role of IL-19 in the immune response is not well understood. IL-19 is recognized and signals through IL-20Rα and IL-20Rβ [37]. Some authors have questioned the IL-19 role in regulating immune cells due to undetectable IL-20Rα chain expression on freshly isolated leukocytes and differentiated macrophages or dendritic cells [37], [38]. Other authors have shown that Th2 cell lines, but not Th1 cell lines, had detectable mRNA levels of IL-20Rα [39]. In addition, several studies have described IL-19 effects on T cells and monocytes [5], [7], [8], [9], [40]. Our findings support these latter observations and suggest that exogenous IL-19 has an anti-inflammatory effect on immune cells from HC. We showed that IL-19 decreased TLR4-induced TNFα production in PBMC cultures from HC but not from active CD patients. Like other authors, we also found that long exposure to IL-19 has two kinds of effects on HC T cells [4], [8]. First, it increased expression of IL-4 but not IFNγ, suggesting that IL-19 is capable of inducing Th2 response in T cells. This possibility is supported by the increased levels to IL-19 levels found in asthma and uremia, two Th2 behavior diseases [8], [9]. Second, long exposure of IL-19 significantly increases CTLA4 but not GITR expression on Treg cells (CD4+CD25+CD127−) from HC but not from active CD patients, suggesting that IL-19 influences the Treg phenotype. Yeh Ching-Hua similarly found that rhIL-19 induced a regulatory phenotype (FOXP3) and regulatory function on CD4 T cells from HC [7]. Taken together, these results show that IL-19 acts differently on PBMC from HC and active CD patients.

Several hypotheses could explain the different response of cells from active CD patients and HC to IL-19. One explanation is that monocytes from active CD patients and HC might express distinct protein levels of IL-20Rα and/or IL20Rβ. However, at the mRNA level, we observed that monocytes from active CD patients and HC expressed similar but low levels of IL-20Rα controls (expression level as mean ± SD: 3.07±0.11 and 3.10±0.03, respectively). Furthermore, published results showed undetectable levels of IL-20Rα by PCR [37], [38], [41]. No results have been published about expression of IL-20Rβ on the cell surface of mononuclear cells from peripheral blood. In view of its sensitivity and unicellular analysis, flow cytometry will likely be the final demonstration of IL-20R protein expression on the surface of monocytes and T cells. Unfortunately, monoclonal IL-20R antibodies for cytometry are not currently available.

Another explanation for the different IL-19 response in active CD patients may be altered protein expression or activity for downstream IL-20R molecules (STAT3, STAT1, TyK and JAK1, etc) or different affinity for exogenous IL-19 by IL-20R. However, other groups showed that a short exposure to IL-19 failed to phosphorylate STAT3 in mononuclear cels from peripheral blood [40]. In view of these results, we cannot rule out that IL-19 could signal through another pathway in immune cells.

Our findings showed that IL-19 was regulated not only by IL-10 but also by other TLR4 induced factors. In this regard, addition of rhIL-10 to cell cultures inhibited IL-19 protein production by monocytes from active CD patients and HC during TLR4 and TLR2 activation. Previous reports have shown that IL-10 decreased IL-19 expression in unstimulated cultures at mRNA level [5]. Inversely, blocking IL-10 induced the expression of IL-19 by monocytes activated by TLR2 but not by TLR4 ligands. This observation suggests that activation of TLR4 leads to the expression of other unknown factors controlling IL-19 production by monocytes from active CD patients and HC.

Although IL-19 and IL-10 are both members of the IL-10 family, we found that the two cytokines had different effects on monocytes. IL-19 only decreased TNFα production via TLR4 in HC. However, IL-10 diminished the production of TNFα via TLR4 and via TLR2 in HC and active CD patients. PBMC from active CD patients were then able to respond to IL-10 but not to IL-19.

In conclusion, we have shown that IL-19 can work as a regulatory cytokine in the inflammatory response of immune cells. Our findings suggest that defects in IL-19 expression and the lack of response to this cytokine may contribute to the inflammatory mechanisms described for active CD patients.
